# Safety and Impacts of Physical Activity for Individuals Living With Hypermobility Spectrum Disorders and Hypermobile Ehlers-Danlos Syndrome: Protocol for a Scoping Review

**DOI:** 10.2196/80394

**Published:** 2026-02-04

**Authors:** Donald William Golden, Jonathan L Low, Julia T Daun, C Allyson Jones, Liz Dennett, Susan Nicole Culos-Reed, Ranita Harpreet Kaur Manocha

**Affiliations:** 1 Cumming School of Medicine, University of Calgary, Calgary, AB, Canada; 2 Faculty of Kinesiology, University of Calgary, Calgary, AB, Canada; 3 Department of Physical Therapy, University of Alberta, Edmonton, AB, Canada; 4 Geoffrey and Robyn Sperber Health Sciences Library, University of Alberta, Edmonton, AB, Canada; 5 Department of Clinical Neurosciences, Cumming School of Medicine, University of Calgary, Room AC144, Special Services Building, Foothills Medical Centre, 1403 – 29 Street NW, Calgary, AB, T2N 2T9, Canada, 1-403-944-4224

**Keywords:** Ehlers-Danlos syndrome, exercise, joint instability, safety, International Classification of Functioning, Disability and Health

## Abstract

**Background:**

Although physical activity (PA) participation has known health benefits, many individuals with hypermobility spectrum disorders (HSD) and hypermobile Ehlers-Danlos syndrome (hEDS) have difficulties participating in PA. HSD/hEDS affect approximately 1 in 500 people and are more prevalent in females. HSD/hEDS may result in frequent joint injuries, chronic pain, and generalized fatigue. These symptoms, and a fear of new or reinjury, may result in barriers to PA participation. Overall, there is limited research on PA in this population. Previous exercise reviews have not included structured PA such as sports and occupational activities; unstructured PA such as play, household, or leisure activities; or younger ages, including children. Additionally, some females with HSD/hEDS report experiencing more frequent joint injuries and worsening pain and fatigue during times of hormonal transitions, such as puberty, pregnancy, as well as cyclically across the menstrual cycle. Some females also report improvements in symptoms and a reduction in injury frequency following menopause. The impacts of PA during these times of changing hormone levels for females living with HSD/hEDS are uncertain. A clear understanding of what types of structured and unstructured PA are safe and helpful for individuals of all ages with HSD/hEDS, and if PA should be adapted during times of female hormonal changes, is lacking.

**Objective:**

We propose a scoping review protocol to map and synthesize the evidence regarding considerations that may impact structured and unstructured PA participation in HSD/hEDS for individuals of all ages and during times of female hormonal transitions.

**Methods:**

A scoping review will be conducted using Covidence (Veritas Health Innovation Ltd) and Microsoft Excel (Microsoft Corp) to map the evidence regarding the impacts of PA on safety, physical health, and quality of life. These outcomes will be assessed using the World Health Organization International Classification of Functioning, Disability and Health framework.

**Results:**

The database search was performed on August 22, 2024, and updated on September 8, 2025. Data extraction started in September 2025 and is ongoing. The results are expected to be published by August 2026.

**Conclusions:**

This proposed scoping review will aid in defining critical research directions regarding PA in HSD/hEDS, which may help inform guidelines outlining the risks and benefits of structured and unstructured PA. This review will also help define existing evidence for age-specific and hormone-related considerations regarding the impacts of PA in this population. This is particularly important as PA may help ameliorate the physical and mental symptoms associated with HSD/hEDS and may improve quality of life for these individuals across the lifespan.

## Introduction

### Overview

Hypermobility spectrum disorders (HSDs) and the hypermobile subtype of Ehlers-Danlos syndrome (hEDS) are conditions that predominantly affect females [1]. While the true prevalence of these conditions is unclear and thought to be underestimated [2], the combined prevalence of HSD/hEDS has been reported to be 1 in 500 (Wales, United Kingdom), and recent unpublished data have shown a prevalence of 1 in 227 (Northumberland, United Kingdom) [1,3]. HSD/hEDS may be characterized by recurrent joint injuries, chronic pain, generalized fatigue, and muscular weakness [4]. As a result, many individuals with HSD/hEDS report a fear of new or reinjury due to movement (kinesiophobia), and many tend to be physically inactive [5-7]. Inactivity may further exacerbate HSD/hEDS symptoms [6,7], prompting a vicious cycle of symptoms reinforcing sedentary behavior and vice versa.

Physical activity (PA) is defined as any movement that increases energy expenditure above resting levels [8,9]. PA may be categorized as structured (exercise, sports, and occupational activities) or unstructured (play, household, or leisure activities, and functional daily movements such as walking or climbing stairs) [9]. Exercise is a structured form of PA that is repetitive, structured, and purposeful for improving health or fitness [9]. PA has known health benefits, including improved cardiovascular, bone, and mental health [8]. As the physical and cognitive benefits of PA may improve quality of life in HSD/hEDS [5], a better understanding of optimal guidance and prescription of PA for this population is needed, particularly around the salient risks and tangible benefits of PA.

Females with HSD/hEDS have reported worsening symptoms and more frequent joint injuries during puberty, pregnancy, and at various points in their menstrual cycles [10]. Symptom severity and injury frequency have also been reported to improve after menopause [10]. Changes in symptoms and injury risk during these times may impact an individual’s ability to participate in PA. To our knowledge, there has been no mention of female sex hormones, puberty, the menstrual cycle, pregnancy, or menopause in previous reviews of structured exercise in HSD/hEDS [11-17].

The World Health Organization International Classification of Functioning, Disability, and Health (ICF) is an internationally recognized classification system that provides a conceptual framework to determine outcomes relevant to daily function, including physical, cognitive, social, and environmental domains [18]. An ICF model has been developed specifically for Ehlers-Danlos syndromes based on clinical presentations and patient experiences, revealing that HSD/hEDS impacts each domain of the ICF [5,7]. Therefore, the ICF provides a relevant framework to categorize PA-associated outcomes related to quality of life for individuals with HSD/hEDS.

Guidance as to what PAs are safe, appropriate, and beneficial for living with HSD/hEDS may help reduce pain and fear associated with PAs in this population. Prior reviews have focused on exercise interventions as part of physical therapy treatment in HSD/hEDS, including therapeutic exercise and strength training alongside manual therapy or other modalities for rehabilitation [11-17,19]. However, multiple knowledge gaps remain. Prior reviews did not consider children, older adults, effects of female sex hormones, or times of female hormonal changes during the lifespan, unstructured PA, or any structured activities outside of physical therapy and rehabilitation, such as different sports or occupational activities. Therefore, this scoping review will help identify what PAs are appropriate and safe for individuals with HSD/hEDS outside of a physical therapy session, if PA should be adapted during specific times of female hormonal changes, and how PA across the lifespan may be affected. This will help guide clinicians in PA counseling for symptom management and improved quality of life in this patient population [5].

### Objectives

This protocol will detail the methodology of the proposed scoping review, which will appraise the evidence regarding the safety and impacts of structured and unstructured PA for individuals of all ages with HSD/hEDS. Categorizing the effects of PA in HSD/hEDS based on ICF domains will provide a comprehensive description of the impacts on quality of life. As female sex hormones may influence symptoms of HSD/hEDS, it is also important to consider hormonal changes across the lifespan when assessing the safety and impacts of PA. Based on the state of the literature, a scoping review is justified [20].

### Aims and Research Questions

The aims and research questions are listed in Textbox 1.

Textbox 1. **Aims and research questions.**

**Primary aim: identify all structured and unstructured physical activities (PAs) that have been investigated in hypermobility spectrum disorder (HSD)/hypermobile Ehlers-Danlos syndrome (hEDS). Primary research questions:**

- In HSD/hEDS, what types of PAs have been investigated?

**Secondary aim: describe the extent of evidence related to the benefits and harms of the identified PAs across several ICF domains within the International Classification of Functioning, Disability, and Health (ICF) “Body Functions and Structures” and “Activities and Participation” categories.**

- For each PA identified:

- “Body Function” domain:

- Mental functions: number of studies, benefits, and adverse events (types and frequencies)
- Sensory functions and pain: number of studies, benefits, and adverse events (types and frequencies)
- Functions of the cardiovascular and respiratory systems: number of studies, benefits, adverse events (types and frequencies)
- Functions of the digestive and metabolic systems: number of studies, benefits, and adverse events (types and frequencies)
- Genitourinary and reproductive functions: number of studies, benefits, and adverse events (types and frequencies)
- Neuromusculoskeletal and movement-related functions: number of studies, benefits, and adverse events (types and frequencies)
- Functions of the skin: number of studies and adverse events (types and frequencies)

- “Body Structure” domain:

- Structure of the nervous system: number of studies, benefits, and adverse events (types and frequencies)
- The eye, ear, and related structures: number of studies, benefits, and adverse events (types and frequencies)
- Structure of the cardiovascular and respiratory systems: number of studies, benefits, and adverse events (types and frequencies)
- Structures related to the digestive and metabolic systems: number of studies, benefits, and adverse events (types and frequencies)
- Structures related to movement: number of studies, benefits, and adverse events (types and frequencies)
- Skin structures: number of studies and adverse events (types and frequencies)

- “Activities and Participation” domain

- Mobility: number of studies, benefits, and adverse events (types and frequencies)
- Self-care: number of studies and benefits
- Interpersonal interactions and relationships: number of studies and benefits
- Community, social, and civic life: number of studies and benefits

- “Environmental Factors” domain

- Support and relationships: number of studies and benefits
- Attitudes: number of studies and benefits

**Tertiary aim: within the context of the ICF Body Functions: Genitourinary and Reproduction Functions domain, identify and describe the extent of evidence regarding safety, benefits, or harms of identified PAs in HSD/hEDS in relation to the influence of changes in female sex hormones. Tertiary research questions:**

- How many studies reported on adverse events during PA or the impact of PA along with:

- Puberty status?
- Menstrual cycle phase, length, and regularity, or cessation of menstruation?
- Hormonal contraceptive use?
- Pregnancy?
- Menopause status?

- What were the findings of these studies?

## Methods

### Protocol Design

This proposed scoping review will follow the methodology described by Arksey and O’Malley [20], Peters et al [21], and the PRISMA-ScR (Preferred Reporting Items for Systematic Reviews and Meta-Analyses extension for Scoping Reviews) [22]. Any deviations from this protocol will be included as an update within the OSF (Open Science Framework) registration and a supplementary document in the final review. Ethics approval from an institutional review board was not necessary, as this review will only pursue secondary use of publicly available published data.

### Eligibility Criteria

Eligibility criteria will be defined according to the PCC (population, concept, and context) format [22] (Table 1).

**Table 1.** Inclusion and exclusion criteria.

	Inclusion criteria	Exclusion criteria
Population		
	Participants with any of: hEDS^a^, HSDs^b^, symptomatic generalized joint hypermobility, joint hypermobility syndrome, benign joint hypermobility syndrome, or EDS^c^ type III^d^. Studies that report outcomes of other connective tissue disorders as well as HSD/hEDS will be included; however, extraction will focus on the outcomes of those with HSD/hEDS. Any age, sex, or gender. Any PA^e^.	Solely includes genetically defined connective tissue disorders (ie, Marfan syndrome, Loeys-Dietz syndrome, and vascular EDS^c^). This is necessary as many of these conditions are associated with life- and limb-threatening arterial complications, and as such would have different implications for PA.
Concept		
	PA safety: adverse events such as falls, heart attack, stroke, aortic dissection, respiratory distress, injury (strain, sprain, subluxation, or dislocation), etc. Benefits or harms of PA on quality of life (Table 2).	Multimodal interventions (ie, exercise plus nutritional intervention). Therapeutic modalities for pain (ie, acupuncture or manipulation). Psychotherapeutic modalities (ie, exercise counseling and cognitive behavioral therapy).
Context		
	As the HSD/hEDS nomenclature has undergone reclassification in 1988, 1997, and 2017, no limits will be placed on the timeframe for when studies were published or when data collection occurred. Studies from any geographical location, country, or institution, and studies including participants from any cultural or social background.	Non-English language^f^ Nonhuman participants

a hEDS: hypermobile Ehlers-Danlos syndrome.

b HSD: hypermobility spectrum disorder.

c EDS: Ehlers-Danlos syndrome.

d Multiple terms have been incorporated to reflect changes in nomenclature over time. A clinical diagnosis for any of these conditions must cite or adhere to established diagnostic criteria in use at the time of diagnosis [4,23-26].

e PA: physical activity.

f A Multimedia Appendix will be included of potentially relevant studies in languages other than English.

**Table 2.** Data items and outcomes according to the ICF^a^ domain [5].

Data item	Description
Participant factors	
Diagnosis	Hypermobile Ehlers-Danlos syndrome, hypermobility spectrum disorders, symptomatic generalized joint hypermobility, joint hypermobility syndrome, benign joint hypermobility syndrome, and Ehlers-Danlos syndrome type III.
Comorbidities or associated conditions	POTS^b^, MCAS^c^, fibromyalgia, asthma, etc.
PA^d^ details	Frequency, intensity, and duration. Structured versus unstructured (Structured PA may include exercise, sports, or occupational activities. Unstructured PA may include play, household or leisure activities, or functional daily movements.).
Comparative intervention	No PA, nutrition intervention only, etc.
Adverse events	Occurrence and frequency of falls, strains, sprains, subluxations, dislocations, cardiovascular events, respiratory distress, and syncope.
Impact of PA or inactivity	Defined as helpful or beneficial if outcomes related to specific ICF domains are improved. Defined as harmful if outcomes related to ICF domains are diminished.
Hormonal contraceptive use	If reported, type: intrauterine system (copper or hormone), oral (combined or progestin only, monophasic, biphasic, or triphasic), and other (transdermal, vaginal ring, etc).
Menstrual cycle influences	If reported: menstrual cycle phase, length, regularity, and amenorrhea.
Menopause status	If reported: premenopause, perimenopause, or postmenopause.
Puberty status	If reported: prepubertal, peripubertal, or postpubertal.
Pregnancy status	If reported: first, second, or third trimester.
Physical outcomes	ICF domain
Vestibular and orthostatic symptoms	Body function, sensory functions, and pain. If reported: dizziness, syncope, orthostatic hypertension, etc.
Gastrointestinal and urinary symptoms	Body function, digestive, metabolic, and endocrine systems. If reported: gastroesophageal reflux, heartburn, bloating, recurrent abdominal pain, diarrhea, irritable bowel syndrome, etc.
Deconditioning	Body function, cardiovascular, hematological, immunological, and respiratory systems. If reported: negative changes in muscle composition, cardiovascular function, and aerobic fitness.
Muscle strength	Body function, neuromuscular, and movement-related functions.
Muscle endurance	Body function, neuromuscular, and movement-related functions.
Balance	Body function, neuromuscular, and movement-related functions.
Proprioception	Body function, neuromuscular, and movement-related functions.
Functional performance	Body function, neuromuscular, and movement-related functions.
Exercise capacity	Body function, neuromuscular, and movement-related functions.
Bone health	Body structure and structures related to movement.
Mobility limitations	Activities and participation, general tasks and demands, mobility, and self-care.
Use of technology or assistive devices to enhance participation	Environmental factors, products, and technology. If reported: braces, mobility aids, apps, reminders, etc.
Psychological outcomes	
Fatigue	Body function and mental functions. If reported: general, cognitive, or physical fatigue.
Anxiety	Body function and mental functions.
Depression	Body function and mental functions.
Sleep quality	Body function and mental functions.
Stress	Body function and mental functions.
Kinesiophobia	Body function and mental functions.
Pain	Body function, sensory functions, and pain. If reported: whole body pain, specific joint pain, visceral pain, headache, etc Nociceptive pain and neuropathic pain.
Social outcomes	
Life satisfaction	Activities and participation, and self-care.
Relationship avoidance or satisfaction	Activities and participation, interpersonal relations, and relationships.
Work status	Activities and participation, and major life areas. If reported: full-time, part-time, sick leave, long-term disability, etc.

a ICF: International Classification of Functioning, Disability, and Health.

b POTS: postural orthostatic tachycardia syndrome.

c MCAS: mast cell activation syndrome.

d PA: physical activity.

### Information Sources

Databases to be searched include MEDLINE, Embase, CINAHL, SPORT Discus, and Scopus with a search end date of September 5, 2025. Study designs such as randomized controlled trials, nonrandomized controlled trials, prospective and retrospective cohort studies, case-control studies, cross-sectional studies, qualitative studies (phenomenology, ethnography, qualitative descriptions, etc), and case studies will be included. Systematic reviews and meta-analyses that meet eligibility criteria will be excluded but flagged for citation chaining. Study designs that will be excluded from this review include conference abstracts or proceedings, editorials, commentaries, and gray literature. Citation chaining will be used for the studies included in the data extraction phase.

### Search Strategy

The search strategy was developed in consultation with DWG, CAJ, and RHKM and performed by a health sciences librarian with experience developing search strategies and performing searches for systematic reviews (LD). A preliminary search was conducted in Ovid MEDLINE to refine the keywords and terms used in the search strategy. This involved running the search with the selected MeSH (Medical Subject Headings) terms and text words in MEDLINE and reviewing the results in consultation with all coauthors. We then performed a preliminary screening of 100 papers to further refine the search terms and keywords. The search strategy will include subject headings (MeSH) and text words related to HSD/hEDS and PA such as Ehlers-Danlos, hypermobility, exercise, gymnastics, stretching, dancing, fitness, PA, sports, tai chi, yoga, qigong, running, swimming, walking, activity tracker, step count, pedometer, daily activities, active lifestyle, housework, manual labor, gardening, and yard work. As the tertiary aim is exploratory, there is no need to add additional keywords related to female hormones, as any relevant papers will be identified using the above strategy. The search will be limited to English, and no date limit will be applied. The search strategy will be adjusted for the specific formatting of each database, and results will be uploaded to the Covidence web-based software platform.

### Study Selection

**Figure 1.**
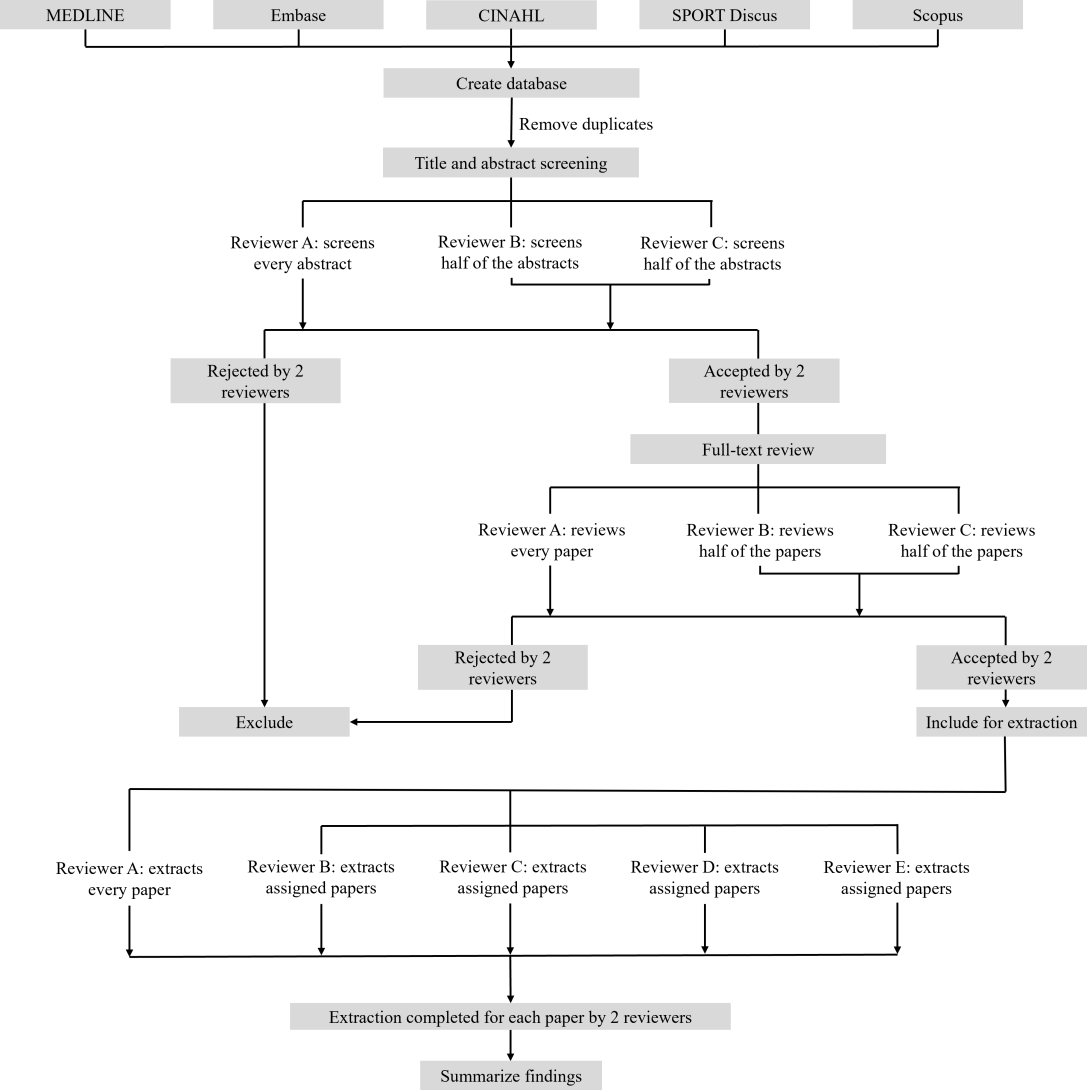
Screening and extraction protocol. Figure created in Microsoft PowerPoint (Microsoft Corp). Figure design adapted with permission from Lima et al [27].

Covidence software will be used to manage the reviews. Three reviewers will independently perform title and abstract screening and full-text review. Reviewer training will involve an educational session outlining the aims, inclusion and exclusion criteria, and screening procedures for each step of the review. Reviewers will be asked to read a reference instructional manual specific to these procedures. Following this, title-abstract calibration will be performed using the first 100 abstracts. Reviewers will independently review the abstracts, and then conflicts and questions will be discussed during a formal debriefing session to ensure consistency between reviewers and consensus in understanding the eligibility criteria. If needed, the instructional manual will be updated. Full-text calibration will be similarly performed through reviewing the first 2 manuscripts assigned to reviewers B and C with reviewer A, who will be reviewing all manuscripts (Figure 1). Again, if needed, the instructional manual will be updated. The screening protocol is shown in Figure 1.

Studies must meet eligibility criteria based on the agreement of 2 reviewers at the title and abstract screening stage, as well as at the full-text review stage, to be included for data extraction. For any studies where 2 reviewers are uncertain after screening the title and abstract (maybe-maybe), these studies will be included for full text review for further assessment against the eligibility criteria. Disagreements between 2 reviewers (yes-no, yes-maybe, or maybe-no) at any stage will be resolved by discussion between all reviewers. Journal titles and original study authors will not be blinded. Interrater agreement for screening and full text review will be reported as Kappa scores.

### Data Extraction

Data extraction will follow the ICF framework. Data items and outcomes according to ICF domains are listed in Table 2. A data extraction template will be created using this framework. Outcomes will be grouped based on the keywords for data items in Table 2. Data extraction will be performed independently by 5 reviewers. Reviewers onboarded for data extraction (D and E) will be trained as described in the screening protocol. Included research studies will be assigned to each of the 4 reviewers (B, C, D, and E), with the fifth reviewer (A) extracting every study to ensure consistency and extraction by the 2 reviewers per paper (Figure 1). Any discrepancies between reviewers will be resolved by consensus discussion between the 5 reviewers. Similar to screening and review, full-text calibration will be performed through extracting the first 2 research studies assigned to each of the 4 reviewers (B, C, D, and E) with the fifth reviewer (A), who will be extracting all included studies. If needed, the instructional manual will be updated. Operational definitions and methods of measurement for each outcome will be extracted and summarized in a table in the final review. Data management will include secure storage of search strategies, the review protocol, reviewer notes, and extracted data in an online Microsoft OneDrive (Microsoft Corp) shared between all authors. The data extraction sheet will be reported as a Multimedia Appendix in the final scoping review. Authors of the original studies will be contacted if information related to the population, concepts, or outcomes is unclear. Three email attempts will be made (once every 2 wk) to the corresponding author to obtain missing information. If no response is received, the missing data will be reported as “not reported” and will not be included in the data synthesis.

### Knowledge Dissemination Plan

Two patient and 4 clinical knowledge users have been involved in the design of this review and the multifaceted integrated knowledge translation plan through formal quarterly meetings. The lived experiences of these knowledge users informed the research questions and specific aims of this review. Specifically, the influence of hormonal contraceptive use on the safety and impacts of PA was included in the research aims based on feedback from these knowledge users. Based on feedback from the knowledge users, the findings of this review will be shared with individuals living with HSD/hEDS, clinicians, and researchers. We will present this at an annual event for patients, researchers, and clinicians and expect attendance to exceed our 2024 event, which was attended by 106 individuals in person and 117 online. We will share the results through a webinar with copresentation with the knowledge users. The knowledge users will also help us craft plain-language summaries of the research findings to be shared on the laboratory websites of the authors. Our research team will generate infographics summarizing the published review to share on social media to reach patients and caregivers. Based on the principal investigator’s laboratory social media metrics, we would expect approximately 300 people to access the infographics. The findings will also be shared with clinicians through 2 local education sessions (1 for physicians and 1 for physical therapists) and 1 provincial education session for physical therapists. Clinicians and researchers will be targeted through presentation at 1 international conference, the publication of this protocol, and the final review.

Findings will be summarized in separate tables related to each research aim. In accordance with the guidelines for scoping reviews [28], quality assessment of methodology and risk of bias will not be performed. Analyses will include frequency counts and descriptive statistics, including percentages or proportions. A narrative summary will be performed to articulate the current state of knowledge regarding the safety, benefits, and harms of PA in HSD/hEDS and patient-specific considerations. This approach has been recommended when including both qualitative and quantitative research designs [29].

## Results

**Figure 2.**
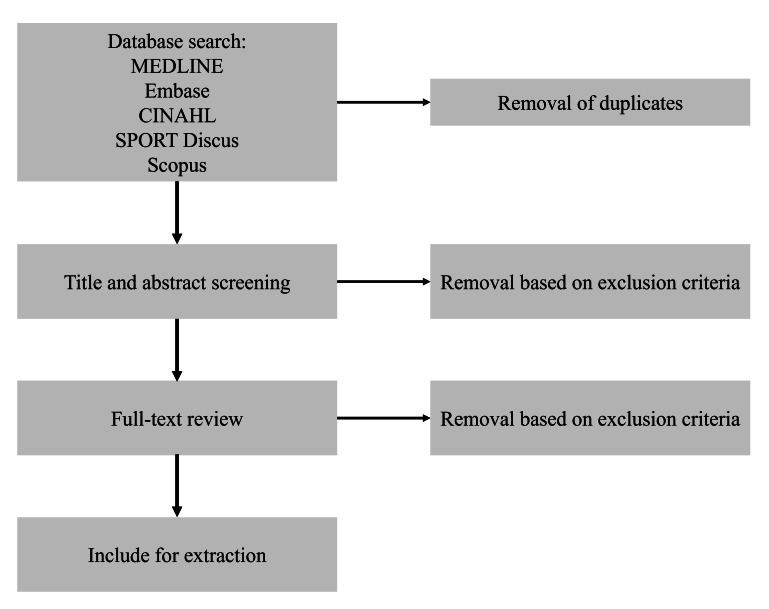
Draft PRISMA flowchart for study selection. Figure created in Microsoft PowerPoint. PRISMA: Preferred Reporting Items for Systematic Reviews and Meta-Analyses.

The search strategy was performed on August 22, 2024, and updated on September 8, 2025. Of 3718 citations identified, 2026 duplicates were removed, 1692 papers were screened, and 57 papers were included for data extraction. Data extraction began in September 2025. The results are expected to be published by August 2026. The number of excluded studies and reasons for exclusion will be reported in the final review using a PRISMA (Preferred Reporting Items for Systematic Reviews and Meta-Analyses) flowchart (Figure 2).

## Discussion

While there is some degree of overlap with previous reviews [11-17,19], this proposed scoping review will add to the understanding of the safety and impacts of PA beyond those included in previous reviews. For example, Brittain et al [19] addressed physical therapy-based exercise, manual therapy, and modalities, which would be the focus of the practicing physical therapist to incorporate during clinical sessions with a patient. Reychler et al [14] also focused on therapeutic exercise as part of physical therapy treatment. In contrast, our review will consider structured PA (exercise and sports) and unstructured PAs (play, household, or leisure activities) that occur outside of physical therapy sessions. Thus, this proposed review may be more practically able to be used by clinicians and patients to explore PA interventions outside of the physical therapist’s office. As such, this proposed review differs in aim and outcomes compared to previous reviews.

Although we expect to find little data on the role of female sex hormones, given the prior literature the authors are aware of [11-17,19], it is important to search for such information and highlight the knowledge gap, if it exists, based on feedback from patient experiences [10]. As HSD/hEDS results in symptoms that can be severely limiting for physical function, and symptoms have been reported to worsen during times of hormonal fluctuations for some females [10], it is important to highlight what literature exists in this area to direct future research around how times of hormonal fluctuations may influence the safety, benefits, or harms of different PAs. To accommodate the expected scarcity of literature, findings related to the tertiary aim will be reported as count data to map existing evidence and clearly define where literature gaps exist.

To promote generalizability of the findings of this review, all age groups will be included, as well as previous classifications of HSD and hEDS. To minimize overlap in the extraction and synthesis for each aim of this review, the research aims are separated based on the specific data items and outcomes, divided into the domains of the ICF, as shown in Table 2. Thus, overlap between aims will be minimized through specific data extraction items and the presentation of results according to each research aim.

While there are many outcomes for extraction in this review, very few studies identified in preliminary screening included more than 5 outcome variables. As such, most studies are expected to not require in-depth extraction of every listed outcome. To acknowledge the burden on reviewers, biweekly check-in meetings will be implemented to make sure reviewers are not overwhelmed. All reviewers will be trained and undergo calibration exercises according to the procedure described in the methods of this protocol. To accommodate the number of outcomes during data synthesis, count and frequency data will be presented in concise tables to summarize the findings separately for each research aim.

To help inform future research and clinical practice, it is critical to understand how outcome measures for various ICF domains are being measured and reported in HSD/hEDS. To account for heterogeneity in the definition and measurement of outcomes between studies, variation in outcome definitions and methods will be summarized in the outcome tables for each research aim during data extraction and synthesis. Such heterogeneous outcomes will be grouped based on the keyword (such as fatigue or functional performance), and the various operational definitions and measurements used by each study will be listed.

The ICF model is not without limitations. The individual experience of HSD/hEDS is highly variable [4,5,30,31], and therefore, some nuance could be missed. In addition, there may be domains specific to HSD/hEDS that are not encapsulated within the typical “Body Function” domains. However, we are exploring multiple ICF domains to better capture the psychosocial-cultural impacts of PA.

Appropriate PA may help ameliorate symptoms and improve the quality of life for individuals living with HSD/hEDS. Defining the current evidence and specific knowledge gaps around structured and unstructured PA outside of physical therapy interventions, as well as age-specific and hormone-related considerations regarding the impacts of PA in this population, is critical to informing specific directions for future research and clinical practice recommendations for safe and potentially beneficial PA in HSD/hEDS. This review serves to provide a comprehensive map of the evidence related to PA in this population and will create a call to action for additional research to address the specific identified gaps, an important step toward the development of recommendations for patients and clinicians. As such, this review will inform future research that may have implications for the development of standardized PA guidelines for individuals living with HSD/hEDS, as well as inform clinicians when creating individualized PA guidelines for patients.

## Abbreviations

hEDS: hypermobile Ehlers-Danlos syndrome
HSD: hypermobility spectrum disorder
ICF: International Classification of Functioning, Disability, and Health
MeSH: Medical Subject Headings
OSF: Open Science Framework
PA: physical activity
PCC: population, concept, and context
PRISMA: Preferred Reporting Items for Systematic Reviews and Meta-Analyses
PRISMA-ScR: Preferred Reporting Items for Systematic Reviews and Meta-Analyses extension for Scoping Reviews
